# The 4-aminopiperidine series has limited anti-tubercular and anti-staphylococcus aureus activity

**DOI:** 10.1186/s12952-015-0024-x

**Published:** 2015-02-13

**Authors:** N Susantha Chandrasekera, Torey Alling, Mai Bailey, Aaron Korkegian, James Ahn, Yulia Ovechkina, Joshua Odingo, Tanya Parish

**Affiliations:** TB Discovery Research, Infectious Disease Research Institute, 1616 Eastlake Avenue E, Seattle, WA 98102 USA; Current address: Seattle Genetics, Bothell, USA

**Keywords:** 4-aminopiperidine, Tuberculosis, Antimicrobial, Staphylococcus aureus

## Abstract

**Background:**

Tuberculosis (TB) caused by *Mycobacterium tuberculosis* is the leading cause of death from a bacterial infection. The 4-aminopiperidine (PIP) series has been reported as having anti-bacterial activity against *M. tuberculosis*. We explored this series for its potential to inhibit aerobic growth of *M. tuberculosis*. We examined substitution at the N-1 position and C-4 position of the piperidine and modifications of the piperidine moiety systematically to delineate structure-activity relationships influencing potency. Compounds were tested for growth-inhibitory activity against virulent *M. tuberculosis*. A selected set of compounds were also tested for its activity against *Staphylococcus aureus*.

**Results:**

The compound with a norbornenylmethyl substituent at the N-1 position and N-benzyl-N-phenethylamine at the C-4 position of the piperidine (**1**) was the only active compound with a minimum inhibitory concentration (MIC) of 10 μM against *M. tuberculosis*. Compounds were not active against *S. aureus*.

**Conclusions:**

We were unable to derive any other analogs with MIC < 20 μM against *M. tuberculosis*. Therefore we conclude that the lack of activity is a liability in this series precluding it from further development.

## Background

Tuberculosis (TB) caused by *Mycobacterium tuberculosis* is the leading cause of death from a bacterial infection. In 2010, according to the World Health Organization (WHO), 8.8 million new cases and 1.4 million deaths from the disease were reported [1]. In addition, more than 2 billion people globally are infected. Of these individuals with latent infection, approximately 10% are expected to develop active TB in their life [2]. Currently the recommended first-line TB treatment regimens require a minimum of 6 months of multidrug therapy, resulting in challenges with patient adherence. The result of inadequate therapy and poor compliance has contributed to a rise in the emergence of multidrug resistant (MDR) and extensively drug-resistant (XDR) strains of *M. tuberculosis* [2-6]. Hence, there is an urgent need to develop novel anti-TB drugs that are effective against both drug sensitive and resistant *M. tuberculosis* [2].

The 4-aminopiperidine (PIP) series was reported as a new class of compounds with activity against *M. tuberculosis* in a high-throughput cell-based assay [7]. Of the 14 compounds reported, 4 gave > 90% inhibition of growth in a single point assay at 10 μg/mL. All of these actives were N-benzyl-N-phenethyl-1-arylpiperidin-4-amines. As a part of our ongoing TB drug discovery program, we were interested in exploring the potential of the PIP series to be developed as a lead series for tuberculosis treatment. We conducted an exploratory structure-activity relationship study to evaluate the series for their activity against *M. tuberculosis* as well as against *staphylococcus aureus*. We tested compound cytotoxicity (TC50) against eukaryotic cells using the Vero cell line (derived from African green monkey kidney cells).

## Results and discussion

We investigated the activity of the series against a virulent strain of *M. tuberculosis* in liquid culture under aerobic growth conditions [8]. First, we probed the consequences of varying the type of substituents at N-1 position of compound **a**, while the substituent at the C-4 position was kept constant. Only the compound with the norbornenylmethyl substituent at N-1 position (**1**) showed good activity with a minimum inhibitory concentration (MIC) against *M. tuberculosis* of 10 μM (Table 1) and is similar to what has been previously reported (4 μM) [7]. However its activity against *Staphylococcus aureus* was > 200 μM. The N-1 substituents comprising of aromatic, heteroaromatic and aliphatic groups were all linked to the piperidine via a methylene spacer. The aromatic substituents we tested include benzyl groups with an electron withdrawing Br group (**8**) and electron donating OMe group. The aliphatics had an acyclic ethyl group (**7**) and a cyclic cyclopentyl group (**10**). The heteroaromatics consisted of two isomeric pyridyl compounds (**4, 5**) and an indole (**3**). All these piperidine compounds had the same substituent at the C-4 position, which is N-(2-phenethyl)-N-(benzyl).

**Table 1:** **Activity of N-substituted analogs of the PIP series against** ***M. tuberculosis***


**Compound**	**R-group**	**Mtb. MIC** ^**a**^ **(μM)**	**% I** ^**b**^	**TC**_**50**_^**c**^ **(μM)**	**Staphylococcus aureus IC**_**90**_ **(μM)**
**1**		10	-	28	>200
**2**	H	>20	<30	14	>200
**3**		20	-	1.8	ND
**4**		>20	<30	31	ND
**5**		>20	<30	21	ND
**6**		>20	74	12.5	ND
**7**		>20	<30	71	>200
**8**		>20	<30	66	ND
**9**		>20	<30	13	ND
**10**		>20	<30	80	ND
**11**		>20	<30	>100	ND

^a^Mtb. MIC is the minimum concentration required to inhibit growth of *M. tuberculosis* completely in liquid culture. MIC is the average of two independent experiments; ^b^% inhibition of growth at 20 μM for compounds with MIC >20 μM.

ND: not determined; ^c^TC_50_ is the concentration required to inhibit growth of Vero cell line by 50%. TC_50_ is the average of two independent experiments.

Alternative substituents at N-1 position as replacement for norbornenylmethyl group gave no improvement on compound activity. In fact all these compounds (Table 1) had MICs above 20 μM, except an indole derivative (**3)**, with an MIC of 20 μM. The benzyl derivative (**6**) also showed some activity achieving >70% inhibition of Mtb. growth at 20 μM with all other compounds demonstrating no activity (<30% inhibition of growth at 20 μM). Compounds **3** and **9** had previously been reported as having anti-tubercular activity with MIC of 9.4 μM and 7.4 μM respectively [7]. However, in our assay the MICs were 20 μM and > 20 μM respectively (Table 1). None of the compounds showed any activity against *S. aureus*.

We then investigated the effect of having a broad range of substitutions at the C-4 position of the piperidine ring and piperidine replacements. The biological activity was determined, but none of the compounds were active, with no inhibition, even at 20 μM (Table 2). The replacement of the phenethyl group in compound **1** by ethyl (**22**) resulted in complete loss of activity, indicating a requirement for flexible hydrophobic group at this position. Similarly, other replacements of the N-phenethyl-N-benzylamine we tried had a negative impact on the biological activity. In addition we investigated effect of change from piperidine to piperazine or morpholine scaffold. However, all these changes were detrimental to anti TB activity of these compounds. Again, none of the compounds showed any activity against *staphylococcus aureus*.

**Table 2:** **Activity of piperidine analogs with a carbonyl group of the PIP series against** ***M. tuberculosis***


**Compound**	**R-group**	**Mtb. MIC** ^**a**^ **(μM)**	**% I** ^**b**^	**TC**_**50**_^**c**^ **(μM)**	**Staphylococcus aureus IC**_**90**_ **(μM)**
**12**		>20	<30	>100	>200
**13**		>20	<30	>100	>200
**14**		>20	<30	41	ND
**15**		>20	<30	>100	>200
**16**		>20	<30	22	>200
**17**		>20	<30	ND	ND
**18**		>20	<30	>100	>200
**19**		>20	<30	68	>200
**20**		>20	<30	>100	>200
**21**		>20	<30	>100	>200
**22**		>20	<30	>100	>200

^a^Mtb. MIC is the minimum concentration required to inhibit growth of *M. tuberculosis* completely in liquid culture. MIC is the average of two independent experiments; ^b^% inhibition of growth at 20 μM for compounds with MIC >20 μM.

ND: not determined; ^c^TC_50_ is the concentration required to inhibit growth of Vero cell line by 50%. TC_50_ is the average of two independent experiments.

Then, we explored the influence of replacing the bridging methylene spacer with an amide as in compound **23**. Similar amides with different substitutions at the N-1 position and N-4 position are shown on Table 3. Despite the significant changes made at the methylene group and N-1 and N-4 substitutions, there was no improvement in anti-tubercular activity noted (MIC > 20 μM).

**Table 3:** **Activity of N-1 and 4-substituted piperidine analogs of the PIP series against** ***M. tuberculosis*** **and staphylococcus aureus**

**Compound**	**Structure**	**Mtb. MIC** ^**a**^ **(μM)**	**% I** ^**b**^	**TC**_**50**_^**c**^ **(μM)**
**23**		>20	<30	42
**24**		>20	<30	47
**28**		>20	<30	75
**25**		>20	<30	>100
**26**		>20	<30	>100
**27**		>20	<30	37
**29**		>20	42	13
**30**		>20	<30	>100

^a^Mtb. MIC is the minimum concentration required to inhibit growth of *M. tuberculosis* completely in liquid culture. MIC is the average of two independent experiments; ^b^% inhibition of growth at 20 μM for compounds with MIC >20 μM. ND: not determined; ^c^TC_50_ is the concentration required to inhibit growth of Vero cell line by 50%. TC_50_ is the average of two independent experiments.

## Conclusions

We conducted a systematic exploration of the 4-aminopiperidine compound series for its inhibitory activity against *M. tuberculosis*. The compound with a norbornenylmethylene substituent at the N-1 position and N-phenethyl-N-benzyl at the C-4 position of the piperidine (**1**) is a singleton, with a minimum inhibitory concentration (MIC) of 10 μM against *M. tuberculosis*. All the compounds were inactive against *S. aureus*. We were unable to improve the activity in this series, even after significant modifications around the piperidine moiety. On this basis we concluded that the series lacks further potential for drug development.

## Methods

### Determination of minimum inhibitory concentration against *M. tuberculosis* (MIC)

MICs were run as described in [8]; briefly MICs were determined against *M. tuberculosis* H37Rv grown in Middlebrook 7H9 medium containing 10% OADC (oleic acid, albumin, dextrose, catalase) supplement (Becton Dickinson) and 0.05% w/v Tween 80 (7H9-Tw-OADC) under aerobic conditions. Bacterial growth was measured by OD after 5 days of incubation at 37°C. The MIC was defined as the minimum concentration required for complete inhibition of growth.

### Eukaryotic cytotoxicity assay

Cytotoxicity was determined against the Vero African green monkey kidney cell line (ATCC CCL-81). Cells were cultured in Dulbecco’s Modified Eagle Medium (DMEM), High Glucose, GlutaMAX™ (Invitrogen), 10% FBS (Fetal Bovine Serum), and 1x of Penicillin-Streptomycin Solution (100 units/mL of penicillin, 100 μg/mL of streptomycin). Compounds were solubilized in DMSO (dimethyl sulfoxide) and assayed using a 10-point three-fold serial dilution starting at the highest concentration of 50 μM. CellTiter-Glo® Reagent (Promega) was added to 96-well plates after 2 days of incubation at 37°C, 5% CO_2_. Relative luminescent units (RLU) were measured using Perkin Elmer Wallac 1420 Victor2 plate reader. Inhibition curves were fitted using the Levenberg–Marquardt algorithm. Toxic concentration (TC50) was defined as the concentration of compound that gave 50% inhibition of growth.

### Determination of activity against *Staphylococcus aureus*

*S. aureus* ATCC 25923 strain was grown in Mueller Hinton II broth at 35°C overnight. The overnight culture was diluted in fresh broth and used as inoculum for the antimicrobial screen. Working stocks (50X) of test compounds were prepared in DMSO. Each well of the 384-well plate contained 30 μl of the inoculum and 0.6 μl of the 50X compound working stock. Plates were incubated at 35°C for 18 hours and 30 μl of the BacTiter-Glo™ reagent (Promega) was added. Relative luminescent units (RLU) were measured using and normalized to growth control wells (2% DMSO) and expressed as % Growth. IC_50_ values were calculated using the Levenberg–Marquardt nonlinear regression algorithm and defined as the compound concentration that produced 50% of the growth inhibitory response. The IC_90_ value was defined as the compound concentration that produced 90% of the growth inhibitory response [9,10].

### Compound purity analysis

HPLC analysis was conducted on an Agilent 1100 series LC system (Agilent ChemStation Rev.A.10.02; Phenomenex-Luna-C18, 4.8 mm × 150 mm, 5 μm, 1.0 mL/min, UV 254 nm, 214 nm, room temperature) with MeCN/H_2_O (0.05% TFA or HCOOH buffer) gradient elution. HPLC-MS was performed on a Gilson 321 HPLC with detection performed by a Gilson 170 DAD and a Finnigan AQA mass spectrometer operating in electrospray ionization mode using a Phenomenex Gemini C18 150 × 4.6 mm column. All the compounds were purchased from ChemBridge Corporation. Purity was calculated from the LC trace at 214 nm.

**1**: LC-MS (ESI) m/z: 401.1 (M + H)^+^, t_R_ = 0.76 min, purity >99%.

**2**: LC-MS (ESI) m/z: 294.9 (M + H)^+^, t_R_ = 0.64 min, purity >99%.

**3**: LC-MS (ESI) m/z: 423.9 (M + H)^+^, t_R_ = 0.79 min, purity >99%.

**4**: LC-MS (ESI) m/z: 385.9 (M + H)^+^, t_R_ = 0.74 min, purity >99%.

**5**: LC-MS (ESI) m/z: 385.9 (M + H)^+^, t_R_ = 0.73 min, purity >99%.

**7**: LC-MS (ESI) m/z: 323.00 (M + H)^+^, t_R_ = 0.70 min, purity >99%.

**8**: LC-MS (ESI) m/z: 464.8 (M + H)^+^, t_R_ = 3.53 min, purity >99%.

**9**: LC-MS (ESI) m/z: 444.9 (M + H)^+^, t_R_ = 2.79 min, purity >99%.

**10**: LC-MS (ESI) m/z: 363.00 (M + H)^+^, t_R_ = 0.71 min, purity >99%.

**11**: LC-MS (ESI) m/z: 434.9 (M + H)^+^, t_R_ = 4.57 min, purity >99%.

**12**: LC-MS (ESI) m/z: 284.00 (M + H)^+^, t_R_ = 0.55 min, purity >99%.

**13**: LC-MS (ESI) m/z: 271.00 (M + H)^+^, t_R_ = 0.73 min, purity >99%.

**15**: LC-MS (ESI) m/z: 194.00 (M + H)^+^, t_R_ = 0.66 min, purity >99%.

**16**: LC-MS (ESI) m/z: 269.00 (M + H)^+^, t_R_ = 0.74 min, purity >99%.

**17**: LC-MS (ESI) m/z: 275.00 (M + H)^+^, t_R_ = 0.54 min, purity >99%.

**18**: LC-MS (ESI) m/z: 366.00 (M + H)^+^, t_R_ = 0.67 min, purity >99%.

**19**: LC-MS (ESI) m/z: 208.00 (M + H)^+^, t_R_ = 0.66 min, purity >99%.

**20**: LC-MS (ESI) m/z: 235.0 (M + H)^+^, t_R_ = 0.69 min, purity >99%.

**21**: LC-MS (ESI) m/z: 235.00 (M + H)^+^, t_R_ = 0.66 min, purity >99%.

**23**: LC-MS (ESI) m/z: 398.9 (M + H)^+^, t_R_ = 4.21 min, purity >99%.

**24**: LC-MS (ESI) m/z: 404.9 (M + H)^+^, t_R_ = 4.12 min, purity >99%.

**25**: LC-MS (ESI) m/z: 413.9 (M + H)^+^, t_R_ = 4.21 min, purity >99%.

**26**: LC-MS (ESI) m/z: 413.9 (M + H)^+^, t_R_ = 3.99 min, purity >99%.

**27**: LC-MS (ESI) m/z: 456.9 (M + H)^+^, t_R_ = 4.53 min, purity >99%.

**28**: LC-MS (ESI) m/z: 432.9 (M + H)^+^, t_R_ = 4.63 min, purity >99%.

**29**: LC-MS (ESI) m/z: 351.00 (M + H)^+^, t_R_ = 0.71 min, purity >99%.

**30**: LC-MS (ESI) m/z: 323.9 (M + H)^+^, t_R_ = 0.71 min, purity >99%.

## Abbreviations

TB: Tuberculosis
PIP: 4-aminopiperidine
MIC: Minimum inhibitory concentration

## References

[1] World Health Organization (WHO) (2011). Global tuberculosis control. WHO Report 2011. Document. WHO/HTM/TB/2011.16.

[2] Ginsberg A (2010). Drugs in development for tuberculosis. Drugs.

[3] Veluchamy M, Madhavan R, Narayanan S, Rajesh L (2013). KatG gene as a surrogate molecular marker leading to cause drug resistance in Mycobacterium tuberculosis isolates. Am J Infect Dis Microbiol.

[4] Parr JB, Mitnick CD, Atwood SS, Chalco KB, Jaime B, Mercedes C (2014). Concordance of resistance profiles in households of patients with multidrug-resistant tuberculosis. Clin Infect Dis.

[5] Saifullah B, Hussein MZB, Al Ali SHH (2012). Controlled-release approaches towards the chemotherapy of tuberculosis. Int J Nanomedicine.

[6] Singla P, Singh R, Sharma M, Aparna CU (2014). Extensively drug resistant tuberculosis: a mini review. Int J Curr Microbiol Appl Sci.

[7] Ananthan S, Faaleolea ER, Goldman RC, Hobrath JV, Kwong CD, Laughon BE (2009). High throughput screening for inhibitors of *Mycobacterium tuberculosis* H37Rv. Tuberculosis.

[8] Ollinger J, Bailey MA, Moraski GC, Casey A, Florio S, Alling T (2013). A dual read-out assay to evaluate the potency of compounds active against *Mycobacterium tuberculosis*. PLoS One.

[9] Clinical and Laboratory Standards Insitute (2009). Performance Standards for Antimicrobial Susceptebility Testing: Nineteenth Informational Supplement. 2012; M100-S22.

[10] Fan F, Butler B, Riss T, Wood K (2004). Quantitate microbial cells using a rapid and sensitive ATP-based luminescent assay. Promega Notes.

